# An Efficient Deep Learning-Based Skin Cancer Classifier for an Imbalanced Dataset

**DOI:** 10.3390/diagnostics12092115

**Published:** 2022-08-31

**Authors:** Talha Mahboob Alam, Kamran Shaukat, Waseem Ahmad Khan, Ibrahim A. Hameed, Latifah Abd. Almuqren, Muhammad Ahsan Raza, Memoona Aslam, Suhuai Luo

**Affiliations:** 1 Department of Computer Science and Information Technology, Virtual University of Pakistan, Lahore 54000, Pakistan; 2 School of Information and Physical Sciences, The University of Newcastle, Newcastle, NSW 2308, Australia; 3 Department of Data Science, University of the Punjab, Lahore 54890, Pakistan; 4 School of Computer Science, National College of Business Administration & Economics, Lahore 54660, Pakistan; 5 Department of ICT and Natural Sciences, Norwegian University of Science and Technology, 7034 Trondheim, Norway; 6 IS Department, College of Computer and Information Science, Princess Nourah Bint Abdulrahman University (PNU), P.O. Box 84428, Riyadh 11671, Saudi Arabia

**Keywords:** medical imaging, skin cancer, deep learning, disease diagnosis system, healthcare

## Abstract

Efficient skin cancer detection using images is a challenging task in the healthcare domain. In today’s medical practices, skin cancer detection is a time-consuming procedure that may lead to a patient’s death in later stages. The diagnosis of skin cancer at an earlier stage is crucial for the success rate of complete cure. The efficient detection of skin cancer is a challenging task. Therefore, the numbers of skilful dermatologists around the globe are not enough to deal with today’s healthcare. The huge difference between data from various healthcare sector classes leads to data imbalance problems. Due to data imbalance issues, deep learning models are often trained on one class more than others. This study proposes a novel deep learning-based skin cancer detector using an imbalanced dataset. Data augmentation was used to balance various skin cancer classes to overcome the data imbalance. The Skin Cancer MNIST: HAM10000 dataset was employed, which consists of seven classes of skin lesions. Deep learning models are widely used in disease diagnosis through images. Deep learning-based models (AlexNet, InceptionV3, and RegNetY-320) were employed to classify skin cancer. The proposed framework was also tuned with various combinations of hyperparameters. The results show that RegNetY-320 outperformed InceptionV3 and AlexNet in terms of the accuracy, F1-score, and receiver operating characteristic (ROC) curve both on the imbalanced and balanced datasets. The performance of the proposed framework was better than that of conventional methods. The accuracy, F1-score, and ROC curve value obtained with the proposed framework were 91%, 88.1%, and 0.95, which were significantly better than those of the state-of-the-art method, which achieved 85%, 69.3%, and 0.90, respectively. Our proposed framework may assist in disease identification, which could save lives, reduce unnecessary biopsies, and reduce costs for patients, dermatologists, and healthcare professionals.

## 1. Introduction

The number of cancer patients is increasing due to smoking, environmental changes, different types of radiation, viruses, alcohol, diet, and lifestyle [1]. The most common and hazardous type of cancer is skin cancer. Skin cancer can be in the form of unusual swelling of skin cells. Skin cancer is spreading worldwide and is a perilous disease [2]. The recorded new skin cancer case rate in the USA is around 5.4 million a year [3]. According to the WHO, annually diagnosed cases of melanoma have increased by 53%, and its mortality rate will increase in the next decade. The failure of early diagnoses has shown a survival rate of less than 14%, but detecting skin cancer at an early stage can increase the survival rate to 97%, approximately [2,4,5]. The Skin Cancer Foundation reported that the skin cancer problem is continuously growing. The most common type of cancer is non-melanocytic such as basal cell carcinoma and squamous cell carcinoma.

Meanwhile, non-melanocytic skin cancer is the basic form to be found, such as basal cell carcinoma and squamous cell carcinoma [6]. It is found that, in the United States, around 1 million SCC cases and 4.3 million BCC cases are diagnosed each year, which are still said to be underestimated. To improve the survival rate, early diagnoses are a foundation, having a 99% correlation with overall survival, and survival is very poor once the disease has penetrated far enough into the skin [7]. An early skin cancer diagnosis is helpful for cancer treatment, although survival is poor once the disease progresses beyond the skin. Medical specialists currently examine the infected person through visual means with the help of polarised light magnification and dermoscopy [8]. The diagnoses also depend on ethnicity, exposure to the sun, social habits, and the patient’s medical history. Medical procedures for skin cancer diagnosis are time-consuming and very tough due to the processing of biopsied lesions [2,9]. In the revolution of healthcare and medicine, AI-enabled computer-aided diagnostics (CAD) solutions make significant contributions, especially in medical imaging. Medical imaging is now a part of clinical practices such as computed tomography (CT), magnetic resonance imaging (MRI), and ultrasound [10,11,12]. Dermoscopy, or less commonly confocal microscopy, plays a vital role in the in vivo visualisation of lesioned features, allowing for more accuracy in risk stratification in the dermatological field. In various studies, AI-based algorithms exceed clinical performance to detect disease in medical imaging [8,13,14].

Deep learning has recently provided end-to-end applications to identify breast cancer, lung cancer, brain tumours, oesophagal cancer, and foot ulcer skin lesions. Imaging techniques such as dermoscopy, CT, HRCT, and MRI have become helpful in diagnosing cancer and are used to obtain data on skin cancer from patients worldwide [8,15]. Skin imaging is a driving force behind skin lesion images and expert annotations for automated CAD. High-speed internet, computing resources, and reliable cloud storage to maintain and distribute skin cancer datasets have sparked the research interest in AI solutions for skin cancer diagnosis [16,17]. These services can be applied to various computers, platforms, and operating systems to transform them into cutting-edge medical devices. A skilful dermatologist usually follows steps, from observation with the naked eye to dermoscopy followed by a biopsy. However, this time-consuming procedure may lead patients to advance to severe stages [18].

Training a classifier and learning the classes present in the dataset are much harder when the dataset is imbalanced. This means that at least one of the classes present in the dataset is significantly larger than the others. The problem is that the classes with few instances have a low error cost and prior probability. Deep learning algorithms have different learning strategies when trained on imbalanced datasets. Data augmentation is a widely used method to overcome the data imbalance problem. Data augmentation generates additional training data by transforming the input training data. Data augmentation is especially beneficial for medical imaging [16,19]. Even for large datasets such as ImageNet [20], it has been shown that data augmentation can be beneficial for very deep architectures [16]. Additionally, data augmentation allows for an easy way to incorporate prior knowledge about possible unseen data. Possible data augmentation schemes range from simple additive or multiplicative image modifications such as intensity shifts to geometric transformations such as rotation, scaling, and elastic deformation, and synthetic data generation [16,21].

Moreover, the main objective is to achieve accuracy in diagnosis, depending on the clinician’s skills. Additionally, the best accuracy for diagnosing skin cancer is no more than 80%. In addition to these difficulties, we do not have enough skilful dermatologists around the globe. A significant amount of work has been carried out to rapidly develop image analysis algorithms to diagnose skin cancer at early stages and solve the aforementioned problems. Most of these algorithms are parametric, i.e., they require normalised data, but the data nature cannot be controlled. Hence, these methods cannot diagnose the disease accurately.

**Contributions:** This research makes various contributions to skin cancer detection.

- The experiments were performed on the latest dataset. The Skin Cancer MNIST: HAM10000 dataset presents cutting-edge images of the newest advancement in cancer lesion detection. Previous studies employed smaller and noisier datasets that led to less efficient results.
- Available skin cancer datasets are highly imbalanced, where multiple lesion cases severely outnumber other lesion types. This paper presents an efficient and novel deep learning-based skin cancer detector for handling imbalanced skin cancer detection problems. Our results reveal that skin cancer detector performance was significantly improved.
- Preprocessing, such as normalisation, image resizing, and data argumentation, was conducted to eradicate the different biases in the dataset amid various classes.
- The performance of the proposed skin cancer detector was validated with state-of-the-art detectors. The proposed skin cancer detector outperformed existing detectors. The proposed skin cancer detector may assist in disease identification, which could save lives, reduce unnecessary biopsies, and reduce costs for patients, dermatologists, and healthcare professionals.
- The proposed deep learning-based skin cancer detector is high-performance, efficient, time-efficient, and empowered with the latest advancement in deep learning and has the least dependence on feature engineering.

## 2. Literature Review

Popescu et al. [22] presented a system based on the deep learning methodology and collective intelligence. Various CNN-based models were employed on the HAM10000 dataset, which can differentiate skin lesions, including melanoma. They analysed the various CNN models to maintain a weight matrix, and their elements were based on neural network lesion classes. Furthermore, the accuracy of their system increased by about three percent. Srinivasu et al. [23] proposed a deep learning-based model for analysing skin disease detection by combining MobileNet and long short-term memory models. The performance of the proposed hybrid model was also analysed to evaluate the growth of the disease. Its results were compared with other state-of-the-art models such as fine-tuned neural networks and CNNs. The proposed hybrid model achieved an accuracy of 85% on the HAM10000 dataset. Khan et al. [24] presented a deep learning-based model for effectively screening skin disease lesions. They performed the experiments using a mask recurrent neural network (MASK-RNN), and a pyramid network was used with Resnet50 to extract and classify the SoftMax classifier. The proposed method exhibited efficient performance on the HAM10000 dataset. In the study of Huang et al. [25], a lightweight skin cancer detector was proposed to aid first-line medical care based on deep learning. The HAM10000 dermoscopy dataset was employed for the training of the multiclass classification model. Their proposed framework achieved an accuracy of 85.8%.

Khan et al. [26] proposed a multiclass skin lesion classification method using local colour-controlled histogram intensity values (LCcHIVs). Then, saliency was measured using a novel deep saliency segmentation technique that includes a CNN, which consists of ten layers. The heat map converts it into a binary image using the thresholding method. They used an improved moth flame optimisation algorithm to avoid dimensionality to select effective features. These features were used with multiple maximum correlation analyses classified using a kernel extreme learning machine (KELM) classifier. The classification performance was evaluated on the HAM10000 dataset and achieved an accuracy of 90.67%. Karl and Enrique [27] also presented a framework for skin cancer identification. In their framework, transfer learning was applied to the convolutional neural network for plain and hierarchical classification and used to differentiate between seven types of skin lesions. Xing et al. [28] presented a Categorical Relation-preserving Contrastive Knowledge Distillation (CRCKD) that was used as a supervisor of the model. They presented a class-guided contrastive distillation (CCD) module for closer image pairs from the same class as a teacher while separating negative images from different classes. This showed higher intra-class similarity and inter-class variance in teachers’ relational knowledge in a robust and balanced manner. Extensive experiments on the HAM10000 dataset demonstrated the superiority of the CRCKD method.

Saket et al. [29] presented a method for skin cancer identification. Their method employed a better evaluation matrix technique than previous methodologies. They used the MobileNet model for identifying cancer and HAM10000 employing transfer learning, and their method achieved an accuracy of 83.1% for seven classes in the dataset. Ameri [30,31] proposed a deep learning-based model for skin lesion classification. The proposed method was trained on the HAM10000 dermoscopy image dataset to classify the melanoma and non-melanoma lesions. Additionally, the deep CNN method was presented for image classification. Transfer learning-based methods or deep learning-based models eliminate the complex segmentation procedure of feature extraction. Andronescu et al. [32] developed a model for identifying skin cancer using dermatoscopic images. A convolutional neural network (CNN) detected images and patterns. The CNN works through three stages: convolutional layer, pooling layer, and fully connected layer. HAM10000 was utilised, containing 10,015 images, including seven skin lesions. These images were first resized to 90 × 120 pixels. Then, they were normalised. The dataset was divided into three parts: training set, test set, and validation set. The CNN was used with a 3 × 3 kernel size and one stride. A rectified linear unit (ReLU) was used as an activation function. Max pooling with a size of 2 × 2 for each layer was used.

## 3. Methodology

**Figure 1:**
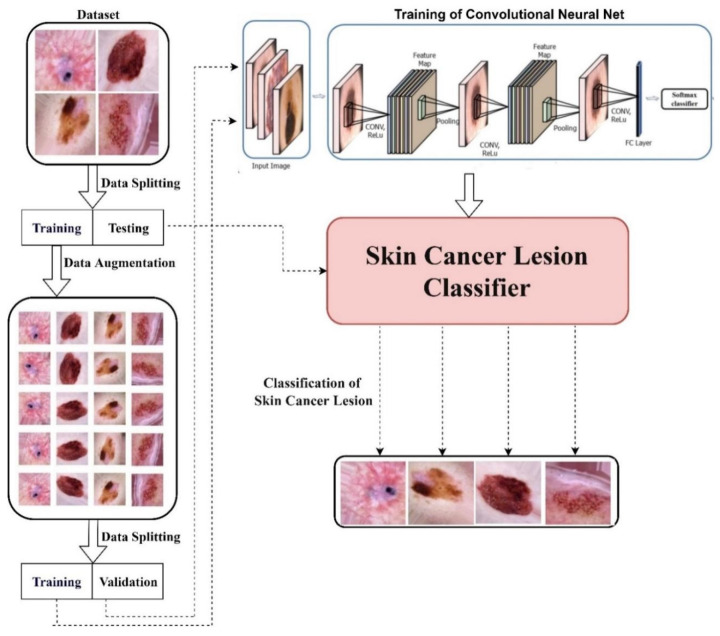
Proposed framework.

First, the skin cancer dataset was obtained for a novel skin cancer detector and divided into training and test sets. Further, augmentation techniques, i.e., rotating and flipping, were applied to the training set to increase the data size to balance the classes. This training dataset was shuffled well and augmented, i.e., reshaped and resized. This balanced dataset was provided to the AlexNet, InceptionV3, and RegNetY-320 models for training. These models were trained with 100% training accuracy. These were tested on the test dataset. Their test accuracies were analysed, performing a comparison of their accuracies. The proposed framework of our study is presented in Figure 1.

### 3.1. Dataset

Meaningful data are an essential component of deep learning. In this study, we used the open-source Skin Cancer MNIST: HAM10000 dataset [33] consisting of 7 types of skin lesions, namely: actinic keratoses and intraepithelial carcinoma/Bowen’s disease (akiec), basal cell carcinoma (bcc), benign keratosis-like lesions (solar lentigines/seborrheic keratoses and lichen planus-like keratoses (bkl)), dermatofibroma (df), melanoma (mel), melanocytic nevi (nv), and vascular lesions (angiomas, angiokeratomas, pyogenic granulomas, and hemorrhage (vasc)). More than 50% of the lesions were confirmed through histopathology (histo). The ground truth for the rest of the cases was either follow-up examination (follow_up), expert consensus (consensus), or confirmation by in vivo confocal microscopy (confocal).

### 3.2. Data Balancing

The HAM10000 dataset was employed in this study, which is prone to highly imbalanced problems. Imbalanced data are a challenging problem while training a deep learning model for a complex task [16,34]. Most deep learning models are designed to work for classes with almost exact data for classification problems. When using a real-time dataset, some events are rare, and we do not have balanced data for each class, especially in the medical domain [35]. This imbalanced dataset often leads to a biased or skewed prediction, affecting the model’s performance. Data augmentation can increase the sample size for those imbalanced classes and produce a balanced dataset [16,21]. Predicting a model trained with supervised deep learning relies on the diversity and the size of the dataset used in training. The relation between a rocket’s engine and the enormous amount of fuel used for a successful mission can represent the relation between the deep learning model and the data size used for training. Generally, deep learning models have many hidden neurons for achieving high performance on complex tasks [36].

**Figure 2:**
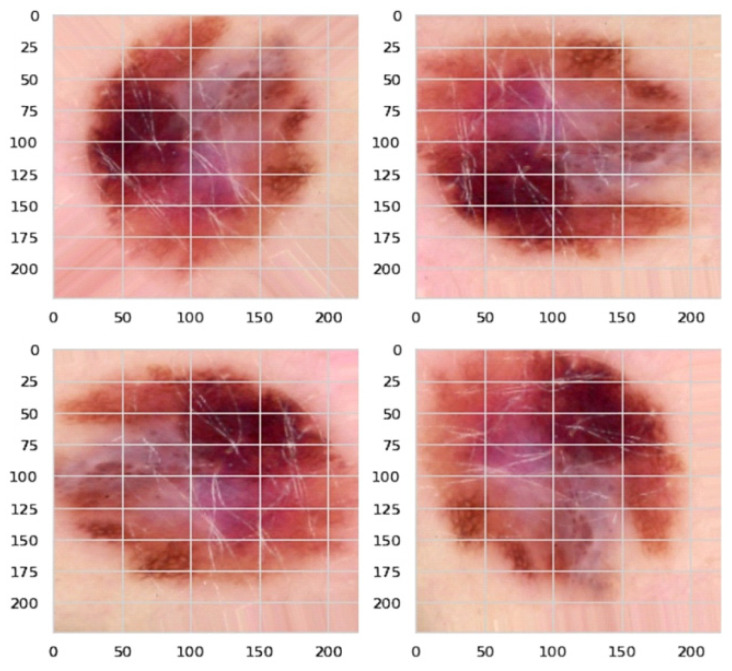
Data augmentation through image rotation.

**Figure 3:**
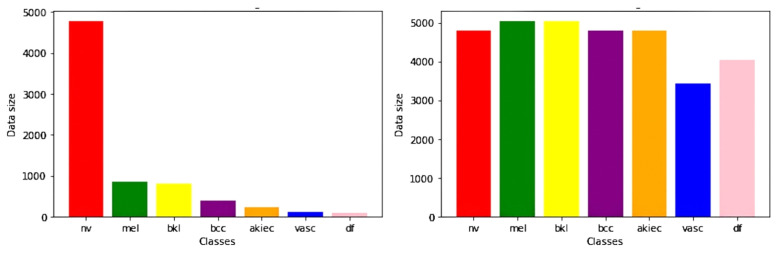
Class distribution before and after data augmentation.

The number of trainable parameters in a deep learning model depends on the number of hidden neurons [37]. Hence, they need a large amount of data with huge diversity for training purposes [38,39]. Data augmentation has been used to address these issues, i.e., increasing the training dataset’s size and diversity. For one class, it has 5000 images, while for another class, it has just a few hundred images. Therefore, this may lead to insufficient training of our model. Hence, we used augmentation techniques such as image rotation to balance our data, as shown in Figure 2. We used data augmentation to increase our dataset’s size by more than 30,000 and to make it balanced for each class. This was done by randomly cropping 256 × 256 patches, flipping the images horizontally, and rotating them at different angles. We then obtained more than 30,000 images for our training set with around 4000–5000 images for each class. Figure 3 shows the distribution of classes before and after the data augmentation.

### 3.3. AlexNet

The first CNN that became famous was AlexNet [40,41,42], which won the 2012 ILSVRC (ImageNet Large-Scale Visual Recognition Challenge), a prestigious challenge in the machine learning field. It was the first architecture that proved the power of CNNs in the context of pattern recognition, becoming the state of the art in image classification, object detection, object recognition, and human pose estimation. AlexNet has eight weight layers, five convolutional layers (where the ReLU per unit follows the convolution operation), and three fully connected layers. The last is a SoftMax layer that returns the probability of belonging to a certain image class. This is an innovative ordering of operations, as in the previous famous network, LeNet, a convolution was always followed by the non-linearity and pooling, not by another convolution. The network has two parallel pipelines executed in different GPUs to speed up the process. It is observed that the first convolution layer uses a filter with a receptive field of 11 × 11, with stride 4 (number of pixels the filter shifts from left to right and from up to down), immediately reducing the image spatially. The receptive field diminishes to go deeper into the network to 5 × 5 and finally 3 × 3. This means that the network initially tries to capture statistics for each pixel in a wider region. As the filter size decreases, the image is down-sampled by max pooling operations, whereas the number of filters increases from 96 to 256 and then 384. Thus, the data are compressed spatially and up-sampled in depth. The model has many weights and memory needed for keeping the feature maps during the forward/backward passes. The convolutional part of the network requires more memory but less computation. The fully connected layers have millions of weights, being the most computationally intensive part of the flow.

Two more novel properties are deployed in AlexNet: the ReLU activation instead of tanh, and the local response normalisation. AlexNet empirically shows that training with non-saturating non-linearity is faster and reaches a better convergence point. ReLUs do not necessarily need input normalisation since, for learning to happen, it is enough that some training examples have a positive input. However, using local normalisation helps generalisation. The normalised response is defined for a unit obtained by applying the filter to the position, defining the window size used for normalisation. Lastly, the network is robust to some transformations by exposing it to an augmented dataset (flipped, translated, reflected images where the label is preserved) and addressing overfitting by applying dropout in the fully connected layers. Being the first successful deep network, the representation properties of AlexNet have been studied extensively. There was already an understanding that invariance and abstraction of features are created as we move deeper in a network; the first layers in a convolutional network represent Gabor features. The higher ones correspond to complex concepts in the image.

### 3.4. InceptionV3

InceptionV3 [43] is an updated version of GoogleNet [44], also called InceptionV1, which reduces the number of parameters concerning state-of-the-art models 12 times. The first version of the Inception architecture was introduced as GoogleNet in 2015. The Inception module applies to different convolutions and max pooling to the same input simultaneously to obtain multi-level features and combines them at the end of the module. To compute them, GoogleNet uses three different filters of sizes 1 × 1, 3 × 3, and 5 × 5. Furthermore, filter blocks were introduced to reduce dimensionality. It has also been noticed that there was a problem of internal covariance shift, which means that when data flow through the network, weights and parameters change data values, which could result in being too big or too small. Sergey et al. [43] introduced batch normalisation, which normalises data after each batch to overcome this problem. This new version of GoogleNet is called InceptionV2. To scale the network, the 5 × 5 convolutional layer was factorised into two consecutive 3 × 3 convolutional layers, and a new version of the network called InceptionV3 was created.

Moreover, the architecture was re-factored to add factorisation convolution, modify the auxiliary classifier, and introduce an efficient grid size reduction and the InceptionV3 version. The factorisation convolution reduces the number of parameters without decreasing the network efficiency. The factorisation techniques used in InceptionV3 are as follows.

**Factorisation into smaller convolutions**: This technique increases the number of convolutional layers by stacking them to reduce the kernel size for each layer. For example, one layer with a 7 × 7 kernel filter dimension has 49 parameters, while three layers with 3 × 3 have 27. The number of parameters is reduced by 45%. With the usage of this technique, it is possible to modify a single Inception module (basic structure of the InceptionVX architectures) and reduce the number of network parameters.

**Factorisation into asymmetric convolutions**: This technique reduces the number of parameters using asymmetrical convolutional layers. The main concept is replacing an NxN filter with two consecutive layers of sizes 1 × N and N × 1, usually greater than 2N. For example, one layer with a 7 × 7 kernel filter dimension has 49 parameters, while two layers with 1 × 7 and 7 × 1 have 14 parameters. The number of parameters is reduced by 72%. With the usage of this technique, it is possible to modify a single Inception module and reduce the number of network parameters. The auxiliary classifier, already present since InceptionV1, had some modifications in InceptionV3. The V1 version has 2 auxiliary classifiers, while the V3 version has only 1 auxiliary classifier on top of the last 17 × 17 layers. The purpose of the auxiliary classifier is also different: firstly, it is used to allow for a deeper network; with the V3 version, it is used to regularise the network. Usually, a max pooling layer is added to reduce the number of weights. Sometimes, this layer is not efficient if inserted before a convolutional layer, or it is too expensive if inserted after a convolutional layer. The efficient grid size reduction technique reduces these problems. It creates a hybrid situation. Each layer concatenates a convolutional layer and a part of the max pooling layer.

### 3.5. RegNetY-320

**Figure 4:**
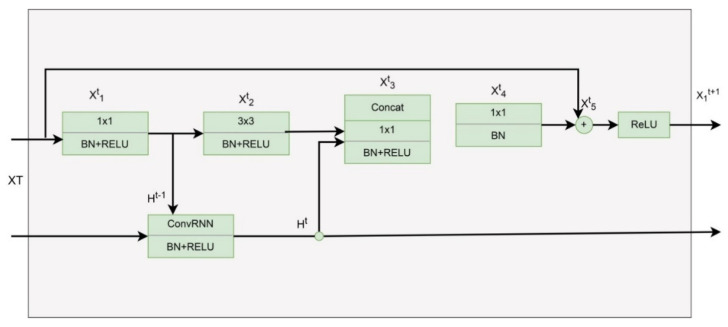
RegNetY-320 architecture.

ResNet and its different versions have performed brilliantly in various computer vision tasks. ResNet was a game-changer because it allowed us to train extraordinarily deep neural networks with more than 150 layers effectively. Figure 4 depicts the bottleneck RegNet module based on the bottleneck ResNet building block proposed to handle a large-scale image.

## 4. Results

The retraining of the deep learning models was performed on an Intel i5 3.0 GHz. The framework chosen for this work was TensorFlow, a deep learning library written in Python and developed by Google. When performing the first stage of training, only original images were used. The oversampled images were added to the dataset in the second stage. In addition to the training images, approximately 3000 (adjusted as a percentage of the total input images) were used as the test set, regardless of the training set size. The test set was only used at the end of each training session to evaluate the final accuracy of the network. All images, both for training and testing, were randomly sampled from the dataset. Most of the hyperparameters were set to their default values. The exception was the learning rate. The learning rate is probably the most important hyperparameter to change if there is a time constraint (i.e., when exhaustive parameter testing is not an option). When fine-tuning a network, the learning rate should be decreased. Hence, it was changed from the default of 0.01 to 0.001.

The data from each class were split into test and training sets. The weightage for the test and training sets was almost 30% and 70% for balanced and imbalanced datasets. The images were resized for each model. Training images were rescaled to 1/255 with a batch size of 100 images. HAM10000 has various skin cancer images of imbalanced classes with 10,000 images, including seven types of skin lesions. The first experiment employed the AlexNet, InceptionV3, and RegNetY-320 models on imbalanced data. The characteristics of the CNNs’ architectures employed in the proposed framework are presented in Table 1.

**Figure 5:**
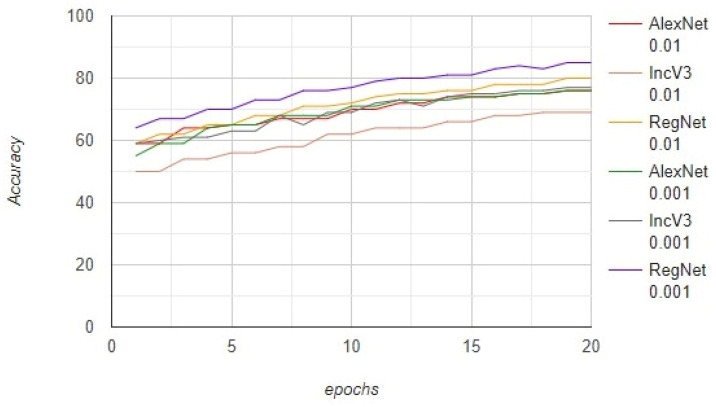
The comparison between the AlexNet, InceptionV3, and RegNetY-320 models on the imbalanced dataset.

The models were trained on the data of 7000 images and tested on 3000 images. The number of epochs was no more than 20, with a batch size of 100. We further trained the models by steepening the learning rate. The AlexNet, InceptionV3, and RegNetY-320 models were trained with a learning rate of 0.01 and achieved an accuracy of 76%, 69%, and 80%, respectively. These models were also trained with a learning rate of 0.001 and achieved an accuracy of 76%, 77%, and 85%, respectively. Furthermore, the AlexNet, InceptionV3, and RegNetY-320 models were trained with a learning rate of 0.01 and achieved an F1-score of 52.2%, 49.9%, and 65.0%, respectively. These models were also trained with a learning rate of 0.001 and achieved an F1-score of 60.2%, 63.7%, and 69.3%, respectively. The results show that the performance of RegNetY-320 significantly increased when the learning rate was changed. The complete results on the imbalanced dataset are presented in Figure 5.

The results obtained using the imbalanced dataset are not efficient. Therefore, a second experiment was performed by employing image augmentation to obtain better results. The various configurations of the image augmentation method are presented in Table 2.

**Figure 6:**
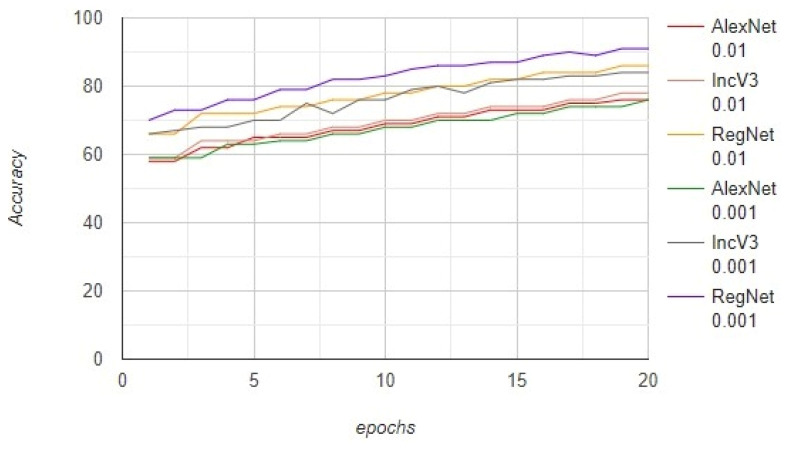
The performance of the proposed framework based on the accuracy.

The size of the images was increased to 32,000 from 10,000 when image augmentation was applied. The models were trained on 22,000 images and tested on 10,000 images. As the dataset was increased, the models could be trained better. The AlexNet, InceptionV3, and RegNetY-320 models were trained with a learning rate of 0.01 and achieved an accuracy of 76%, 78%, and 86%, respectively. These models were also trained with a learning rate of 0.001 and achieved an accuracy of 76%, 85%, and 91%, respectively. Furthermore, the AlexNet, InceptionV3, and RegNetY-320 models were trained with a learning rate of 0.01 and achieved an F1-score of 68.5%, 72.0%, and 78.3%, respectively. These models were also trained with a learning rate of 0.001 and achieved an F1-score of 60.2%, 77.1%, and 88.1%, respectively. The results show that the performance of RegNetY-320 significantly increased when the learning rate was changed. The complete results obtained using the proposed framework are presented in Figure 6.

It can be observed that the results of our proposed framework outperformed the state-of-the-art methods, as shown in Table 3. We employed a data augmentation technique to balance the dataset in our proposed framework. Neural network-based architectures are trained much better on balanced data than imbalanced data. However, we cannot find balanced data in the real world, so we balanced the data using data augmentation. Previous studies claimed that clear convergence is expected to be revealed when training a classifier increases the input data, while Table 3 supports our claim that there is a clear difference between the balanced and imbalanced dataset results.

When analysing the problems with different algorithms, we often need to compare the efficiency of each algorithm to determine which to choose. The ROC curve represents the false positive rate (FPR) and true positive rate (TPR) under different threshold settings. Each graph point represents T and FPR under a specific probability threshold. The threshold ranges from 0 to 1. This is because FPR ranges from 0 to 1, as is obvious from its formula. The ROC curve lies on (0,0) and (1,1) regardless of which model it is. The ideal TPR is 1, which means a specific threshold exists where all positives are labelled as positives. The ideal FPR is 0, which means a specific threshold exists where none of the negatives are labelled as positives. Thus, (0,1) is the ideal point.

The advantage of the ROC curve is that it considers the balance of positive and negative observations. TPR focuses on positive cases, and FPR focuses on negative cases. Therefore, the ROC curve is a more balanced evaluation method. TPR and FPR, the two indicators in the ROC curve, do not depend on a specific category distribution. Therefore, the ROC curve has an outstanding feature compared with other evaluation methods. When the rate of positive and negative observations in the test dataset changes, the ROC curve can remain unchanged. In actual datasets, class imbalance often occurs. There are many more negative observations than positive observations, and vice versa. The distribution of positive and negative observations in the test dataset may also change. The ROC curve can show good stability in this situation. The ROC curve was evaluated both on imbalanced data and the proposed framework. In the case of an imbalanced dataset, the AlexNet, InceptionV3, and RegNetY-320 models were trained with a learning rate of 0.01 and achieved an ROC curve value of 0.83, 0.75, and 0.85, respectively. These models were also trained with a learning rate of 0.001 and achieved an ROC curve value of 0.83, 0.84, and 0.90, respectively.

**Figure 7:**
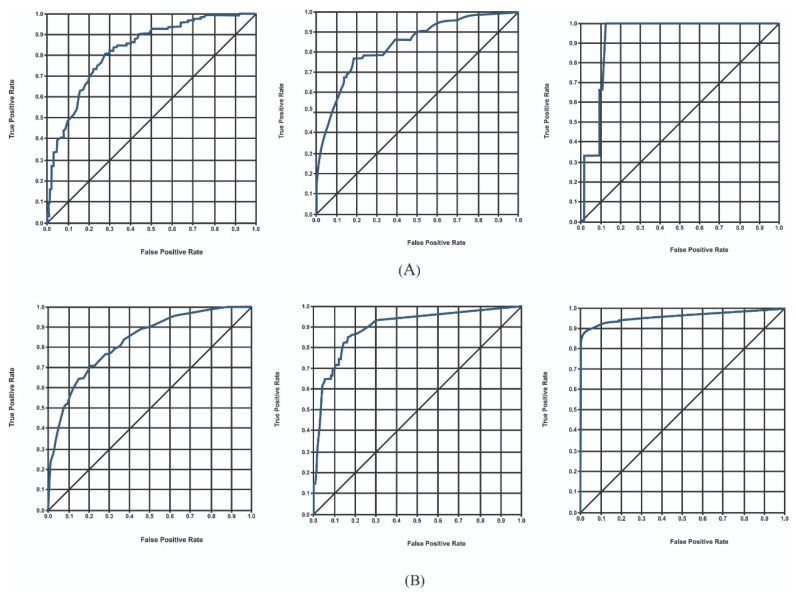
(**A**) ROC curve obtained using AlexNet, InceptionV3, and RegNetY-320 with a learning rate of 0.01; (**B**) ROC curve obtained using AlexNet, InceptionV3, and RegNetY-320 with a learning rate of 0.001.

In contrast, using the proposed framework, the AlexNet, InceptionV3, and RegNetY-320 models were trained with a learning rate of 0.01 and achieved ROC curve values of 0.83, 0.84, and 0.92, respectively. These models were also trained with a learning rate of 0.001 and achieved an ROC curve value of 0.83, 0.89, and 0.95, respectively. The accuracy of the models concerning each class is presented in Table 4. The results show that the performance of the models significantly increased using the proposed framework-based ROC curve. The complete results obtained using the proposed framework based on the ROC curve are presented in Figure 7.

## 5. Discussion

The accuracy achieved on the HAM10000 imbalanced dataset with RegNetY-320 was 85%, while the performance improved to 91% after the proposed framework was employed. Because the size of images also increased from 10,000 to 32,000 images, it was also concluded that the performance can be increased by increasing the dataset size. Furthermore, neural network-based architectures performed better on a balanced dataset for classification problems. Hence, the performance of models is directly proportional to the size of the dataset. The results obtained using ResNet are better than those of AlexNet and InceptionV3. The number of trainable parameters in AlexNet is 200,132,679, leading to an accuracy of 76%. This adds more evidence to the accuracy of the number of trainable parameters in the neural network. However, when we trained InceptionV3 with just 22,126,759 trainable parameters, we showed an unexpected behaviour with an accuracy of 78%. This exception shows that the accuracy depends on the number of parameters. Still, it is more dependent on the architecture of the network, i.e., the sequence of layers, number of convolutional layers, number of connected layers, and the pattern they are connected in. When the learning rate of RegNetY-320 was changed from 0.01 to 0.001, its accuracy increased from 86% to 91% in 20 epochs with a batch size of 100. This shows that it might be evident that the accuracy increases with a decreasing learning rate, or there is still vacant space in this network for more learning and better accuracy. When we changed the learning rate of AlexNet from 0.01 to 0.001, its accuracy improved by fractions, showing that a model with a slower learning rate can extract more features and information from the dataset.

The performance shown by AlexNet, InceptionV3, and RegNetY-320 after training on the imbalanced dataset was not better than that of the proposed framework, even at the same learning rate of 0.001, with an epoch size of 20 and a batch size of 100. The accuracies of AlexNet, InceptionV3, and RegNetY-320 after utilising the proposed framework were 76%, 85%, and 91%, respectively, but decreased to 76%, 77%, and 85% after training on the imbalanced dataset. Certain factors involve a significant decrease in the performance of models. One of those reasons is that the dataset generated using the proposed framework is much larger than the imbalanced dataset. The model can extract more features from a larger dataset than it could with a smaller dataset. Secondly, larger data have more than the model can learn, which is not the case with a smaller dataset. Due to skewed datasets in a classification problem, the interest of the model builds higher towards classes with more data and lower classes of a low data size. In classification problems, the model has to draw boundaries between classes. If the model does not have enough data to differentiate between classes, it starts confusing class boundaries, decreasing its performance [16,21,45]. A comparison of previous studies on the HAM10000 dataset is presented in Table 5.

The results also show that the deep learning-based models performed better on a balanced dataset than on an imbalanced dataset. This might be due to the neural network’s convolutional layers, weight updates, and deep learning. As the neural network does not need pre-extracted features to be fed to the machine learning algorithm but extracts its features based on exciting aspects of the class in the images, it might extract features that are performing well in the dataset, making it more flexible, instead of extracting features that perform well overall, which leads to overfitting [46,47,48,49]. It cannot be verified or falsified whether the deep learning models were overfitted on this dataset, as the classifiers were not tested on other datasets. The generalisability of the classifiers trained on this dataset is unknown. The proposed framework should be generalised to similar tasks and datasets of the same level of complexity. The demonstrated results depend on the dataset, which indicates the biased behaviour of the proposed framework. The generalisation of the proposed framework is indeed a limitation of our work.

**Table 5:** Comparison of previous studies on the HAM10000 dataset.

Reference	Year	Models	Accuracy (%)	F1-Score (%)
[50]	2022	DenseNet201	82.9	74.4%
[51]	2022	Wide-ShuffleNet	86.3	____
[22]	2022	Collective Intelligence-based System	86.7	____
[23]	2021	MobileNet V2-LSTM	90.7	____
[24]	2021	Mask-RCNN	86.5	86.2
[25]	2021	EfficientNet-B4	85.8	____
[26]	2021	Kernel extreme learning machine (KELM) classifier	90.6	____
[27]	2021	DenseNet201	87.7	85.5
[28]	2021	CRCKD algorithm	85.6	76.4
[29]	2020	MobileNet	83.1	83.0
[30]	2020	Deep CNN	84.0	____
[32]	2019	L2 regularisation	72.1	____
**Our study**	**2022**	**Proposed framework**	**91.0**	**88.1**

## 6. Conclusions

Skin cancer is one of the deadliest diseases globally if not detected at the early stages. Many deep learning-based applications using computer vision are designed to assist in detecting skin cancer. This paper sought to find a solution for classifying skin lesions using images with an efficient performance. A novel framework was proposed to solve the problem of data imbalance. The classes in the dataset were not balanced, limiting the performance of deep learning models. Data augmentation techniques are used to increase the size of the dataset and resolve the data imbalance issue. Our proposed framework was trained on the Skin Cancer MNIST: HAM10000 dataset. AlexNet, InceptionV3, and RegNetY-320-based deep learning models were trained on balanced and imbalanced datasets. The proposed framework was tuned on different hyperparameters, i.e., the learning rate, epochs, and batch size in which the learning rate was changed, but the epochs and batch size were fixed. The performance of the RegNetY-320 model was better than that of AlexNet and InceptionV3 in terms of the accuracy and ROC curve both on the imbalanced and balanced datasets.

Furthermore, the accuracy obtained using the proposed framework was 91%, which was significantly better than the state-of-the-art method, which achieved 85%. In the future, to see a convergence in the accuracy of RegNetY-320, it would be valuable to test it on a larger training set. It would be interesting to compare the results of the proposed framework with those of dermatologists for the clinical implementation of our proposed framework in skin cancer identification. This would provide healthcare institutions with guidance on when it is appropriate to use our proposed framework as a second opinion or even replace the human factor. Furthermore, the proposed framework should also be tested on other skin cancer datasets.

## Figures and Tables

**Table 1:** Characteristics of the CNNs’ architectures in the proposed framework.

Model	Hidden Layers	Image Size	Parameters	Learning Rate
AlexNet	8	227 × 227	200,132,679	[0.01, 0.001]
InceptionV3	48	299 × 299	22,126,759	[0.01, 0.001]
RegNetY-320	150	256 × 256	145,000,000	[0.01, 0.001]

**Table 2:** Various configurations of the image augmentation method.

Technique	Configuration
Rotation (Random)	[0°, 360°]
Translation (Random)	[−10, 10] pixels
Rescaling (Random)	[1/1.6, 1.6]
Flipping	left to right
Shearing (Random)	[−20°, 20°]
Stretching (Random)	[1/1.3, 1.3]

**Table 3:** The comparison of the performance on imbalanced data and the proposed framework.

Model	Learning Rate		Imbalanced Dataset		Proposed Framework (Balanced Dataset)		
		Accuracy	F1-Score	ROC Value	Accuracy	F1-Score	ROC Value
AlexNet	0.01	76%	52.2%	0.83	76%	68.5%	0.83
InceptionV3	0.01	69%	49.9%	0.75	78%	72.0%	0.84
RegNetY-320	0.01	80%	65.0%	0.85	86%	78.3%	0.92
AlexNet	0.001	76%	60.2%	0.83	76%	60.2%	0.83
InceptionV3	0.001	77%	63.7%	0.84	85%	77.1%	0.89
RegNetY-320	0.001	85%	69.3%	0.90	91%	88.1%	0.95

**Table 4:** The accuracy of the models for each class.

Model	akiec	bcc	bkl	df	mel	nv	vasc	Average Accuracy
AlexNet	57.9	76.2	70.1	67.3	68.0	94.9	98.0	76.0%
InceptionV3	75.6	82.2	80.3	82.4	80.5	95.0	99.0	85.0%
**RegNetY-320**	**80.7**	**84.9**	**88.5**	**89.4**	**94.5**	**99.0**	**100.0**	**91.0%**

## Data Availability

The data will be provided upon reasonable request.

## References

[1] Alam T.M., Khan M.M.A., Iqbal M.A., Abdul W., Mushtaq M. (2019). Cervical cancer prediction through different screening methods using data mining. IJACSA Int. J. Adv. Comput. Sci. Appl..

[2] Tavakolpour S., Daneshpazhooh M., Mahmoudi H. (2017). Skin cancer: Genetics, immunology, treatments, and psychological care. Cancer Genetics and Psychotherapy.

[3] Ferlay J., Colombet M., Soerjomataram I., Parkin D.M., Piñeros M., Znaor A., Bray F. (2021). Cancer statistics for the year 2020: An overview. Int. J. Cancer.

[4] Brunssen A., Waldmann A., Eisemann N., Katalinic A. (2017). Impact of skin cancer screening and secondary prevention campaigns on skin cancer incidence and mortality: A systematic review. J. Am. Acad. Dermatol..

[5] Niino M., Matsuda T. (2021). Age-specific skin cancer incidence rate in the world. Jpn. J. Clin. Oncol..

[6] Ciążyńska M., Kamińska-Winciorek G., Lange D., Lewandowski B., Reich A., Sławińska M., Pabianek M., Szczepaniak K., Hankiewicz A., Ułańska M. (2021). The incidence and clinical analysis of non-melanoma skin cancer. Sci. Rep..

[7] Nikolouzakis T.K., Falzone L., Lasithiotakis K., Krüger-Krasagakis S., Kalogeraki A., Sifaki M., Spandidos D.A., Chrysos E., Tsatsakis A., Tsiaoussis J. (2020). Current and future trends in molecular biomarkers for diagnostic, prognostic, and predictive purposes in non-melanoma skin cancer. J. Clin. Med..

[8] Goyal M., Knackstedt T., Yan S., Hassanpour S. (2020). Artificial intelligence-based image classification for diagnosis of skin cancer: Challenges and opportunities. Comput. Biol. Med..

[9] Chan S., Reddy V., Myers B., Thibodeaux Q., Brownstone N., Liao W. (2020). Machine learning in dermatology: Current applications, opportunities, and limitations. Dermatol. Ther..

[10] Kousis I., Perikos I., Hatzilygeroudis I., Virvou M. (2022). Deep Learning Methods for Accurate Skin Cancer Recognition and Mobile Application. Electronics.

[11] Nawaz M., Mehmood Z., Nazir T., Naqvi R.A., Rehman A., Iqbal M., Saba T. (2022). Skin cancer detection from dermoscopic images using deep learning and fuzzy k-means clustering. Microsc. Res. Tech..

[12] Bechelli S., Delhommelle J. (2022). Machine Learning and Deep Learning Algorithms for Skin Cancer Classification from Dermoscopic Images. Bioengineering.

[13] Phillips M., Greenhalgh J., Marsden H., Palamaras I. (2020). Detection of malignant melanoma using artificial intelligence: An observational study of diagnostic accuracy. Dermatol. Pract. Concept..

[14] Alfi I.A., Rahman M.M., Shorfuzzaman M., Nazir A. (2022). A Non-Invasive Interpretable Diagnosis of Melanoma Skin Cancer Using Deep Learning and Ensemble Stacking of Machine Learning Models. Diagnostics.

[15] Reis H.C., Turk V., Khoshelham K., Kaya S. (2022). InSiNet: A deep convolutional approach to skin cancer detection and segmentation. Med. Biol. Eng. Comput..

[16] Mikołajczyk A., Grochowski M. Data augmentation for improving deep learning in image classification problem. Proceedings of the 2018 International Interdisciplinary PhD Workshop (IIPhDW).

[17] Santos M.A., Munoz R., Olivares R., Filho P.P.R., del Ser J., de Albuquerque V.H.C. (2020). Online heart monitoring systems on the internet of health things environments: A survey, a reference model and an outlook. Inf. Fusion.

[18] Wolner Z.J., Yélamos O., Liopyris K., Rogers T., Marchetti M.A., Marghoob A.A. (2017). Enhancing skin cancer diagnosis with dermoscopy. Dermatol. Clin..

[19] Srinivas C., KS N.P., Zakariah M., Alothaibi Y.A., Shaukat K., Partibane B., Awal H. (2022). Deep Transfer Learning Approaches in Performance Analysis of Brain Tumor Classification Using MRI Images. J. Healthc. Eng..

[20] Russakovsky O., Deng J., Su H., Krause J., Satheesh S., Ma S., Huang Z., Karpathy A., Khosla A., Bernstein M. (2015). Imagenet large scale visual recognition challenge. Int. J. Comput. Vis..

[21] Saini M., Susan S. (2020). Deep transfer with minority data augmentation for imbalanced breast cancer dataset. Appl. Soft Comput..

[22] Popescu D., El-khatib M., Ichim L. (2022). Skin Lesion Classification Using Collective Intelligence of Multiple Neural Networks. Sensors.

[23] Srinivasu P.N., SivaSai J.G., Ijaz M.F., Bhoi A.K., Kim W., Kang J.J. (2021). Classification of skin disease using deep learning neural networks with MobileNet V2 and LSTM. Sensors.

[24] Khan M.A., Zhang Y.-D., Sharif M., Akram T. (2021). Pixels to classes: Intelligent learning framework for multiclass skin lesion localization and classification. Comput. Electr. Eng..

[25] Huang H.W., Hsu B.W.Y., Lee C.H., Tseng V.S. (2021). Development of a light-weight deep learning model for cloud applications and remote diagnosis of skin cancers. J. Dermatol..

[26] Khan M.A., Sharif M., Akram T., Damaševičius R., Maskeliūnas R. (2021). Skin lesion segmentation and multiclass classification using deep learning features and improved moth flame optimization. Diagnostics.

[27] Thurnhofer-Hemsi K., Domínguez E. (2021). A convolutional neural network framework for accurate skin cancer detection. Neural Process. Lett..

[28] Xing X., Hou Y., Li H., Yuan Y., Li H., Meng M.Q.-H. (2021). Categorical Relation-Preserving Contrastive Knowledge Distillation for Medical Image Classification. International Conference on Medical Image Computing and Computer-Assisted Intervention.

[29] Chaturvedi S.S., Gupta K., Prasad P.S. (2020). Skin lesion analyser: An efficient seven-way multi-class skin cancer classification using MobileNet. International Conference on Advanced Machine Learning Technologies and Applications.

[30] Ameri A. (2020). A deep learning approach to skin cancer detection in dermoscopy images. J. Biomed. Phys. Eng..

[31] Khushi M., Shaukat K., Alam T.M., Hameed I.A., Uddin S., Luo S., Yang X., Reyes M.C. (2021). A comparative performance analysis of data resampling methods on imbalance medical data. IEEE Access.

[32] Andronescu A., Nastac D., Tiplica G. Skin Anomaly Detection Using Classification Algorithms. Proceedings of the 2019 IEEE 25th International Symposium for Design and Technology in Electronic Packaging (SIITME).

[33] Tschandl P., Rosendahl C., Kittler H. (2018). The HAM10000 dataset, a large collection of multi-source dermatoscopic images of common pigmented skin lesions. Sci. Data.

[34] Sabanci K., Aslan M.F., Ropelewska E., Unlersen M.F. (2021). A convolutional neural network-based comparative study for pepper seed classification: Analysis of selected deep features with support vector machine. J. Food Process Eng..

[35] Johnson J.M., Khoshgoftaar T.M. (2019). Survey on deep learning with class imbalance. J. Big Data.

[36] Bhimavarapu U., Battineni G. (2022). Skin Lesion Analysis for Melanoma Detection Using the Novel Deep Learning Model Fuzzy GC-SCNN. Healthcare.

[37] Codella N.C., Nguyen Q.-B., Pankanti S., Gutman D.A., Helba B., Halpern A.C., Smith J.R. (2017). Deep learning ensembles for melanoma recognition in dermoscopy images. IBM J. Res. Dev..

[38] Khan A., Sohail A., Zahoora U., Qureshi A.S. (2020). A survey of the recent architectures of deep convolutional neural networks. Artif. Intell. Rev..

[39] Batool D., Shahbaz M., Asif H.S., Shaukat K., Alam T.M., Hameed I.A., Ramzan Z., Waheed A., Aljuaid H., Luo S. (2022). A Hybrid Approach to Tea Crop Yield Prediction Using Simulation Models and Machine Learning. Plants.

[40] Krizhevsky A., Sutskever I., Hinton G.E. (2012). Imagenet classification with deep convolutional neural networks. Adv. Neural Inf. Process. Syst..

[41] Alam T.M., Shaukat K., Khelifi A., Khan W.A., Raza H.M.E., Idrees M., Luo S., Hameed I.A. (2022). Disease diagnosis system using IoT empowered with fuzzy inference system. Comput. Mater. Contin..

[42] Yang X., Khushi M., Shaukat K. Biomarker CA125 Feature Engineering and Class Imbalance Learning Improves Ovarian Cancer Prediction. Proceedings of the 2020 IEEE Asia-Pacific Conference on Computer Science and Data Engineering (CSDE).

[43] Szegedy C., Vanhoucke V., Ioffe S., Shlens J., Wojna Z. Rethinking the inception architecture for computer vision. Proceedings of the IEEE Conference on Computer Vision and Pattern Recognition.

[44] Szegedy C., Liu W., Jia Y., Sermanet P., Reed S., Anguelov D., Erhan D., Vanhoucke V., Rabinovich A. Going deeper with convolutions. Proceedings of the IEEE Conference on Computer Vision and Pattern Recognition.

[45] Ali I.S., Mohamed M.F., Mahdy Y.B. (2019). Data augmentation for skin lesion using self-attention based progressive generative adversarial network. arXiv.

[46] Krois J., Mahmood A. (2021). Generalizability of deep learning models for dental image analysis. Sci. Rep..

[47] Shrestha A., Mahmood A. (2019). Review of deep learning algorithms and architectures. IEEE Access.

[48] Shaukat K., Alam T.M., Ahmed M., Luo S., Hameed I.A., Iqbal M.S., Li J., Iqbal M.A. A Model to Enhance Governance Issues through Opinion Extraction. Proceedings of the 2020 11th IEEE Annual Information Technology Electronics and Mobile Communication Conference (IEMCON).

[49] Shaukat K., Alam T.M., Hameed I.A., Khan W.A., Abbas N., Luo S. A Review on Security Challenges in Internet of Things (IoT). Proceedings of the 2021 26th International Conference on Automation and Computing (ICAC).

[50] Fraiwan M., Faouri E. (2022). On the Automatic Detection and Classification of Skin Cancer Using Deep Transfer Learning. Sensors.

[51] Hoang L., Lee S.-H., Lee E.-J., Kwon K.-R. (2022). Multiclass Skin Lesion Classification Using a Novel Lightweight Deep Learning Framework for Smart Healthcare. Appl. Sci..

